# “At-Home” Photobiomodulation: A New Approach for Bell's Palsy Treatment

**DOI:** 10.1155/2021/5043458

**Published:** 2021-09-13

**Authors:** Carlo Fornaini, Zhao Meng, Elisabetta Merigo, Jean-Paul Rocca

**Affiliations:** ^1^Micoralis Laboratory EA7354, Faculty of Dentistry, University of Nice Sophia Antipolis, 24 Avenue des Diables Bleus, Nice 06357, France; ^2^Group of Applied ElectroMagnetics, Department of Engineering and Architecture, University of Parma, Viale G. P. Usberti 181/A, Parma 43124, Italy; ^3^Department of Stomatology, 2nd Hospital, Gonguong Road 425, Shijiazhuang, Hebei, China

## Abstract

**Objective:**

This report is the first one to describe the possibility to use “self‐administered” photobiomodulation (PBM) for Bell's palsy (BP) treatment.

**Background:**

BP is a peripheral disorder of the facial nerve causing sudden paralysis of unilateral facial muscles, and PBM has been successfully suggested for its treatment without any side effect. This is the first case report where a laser device was successfully used at home by the patient herself to treat BP opening new perspectives on the therapy of this disease.

**Methods:**

This report describes the “at-home PBM” treatment performed on a 15-year-old girl who presented BP consisting of acute pain on the right side of her face, difficulty in biting and dripping saliva from the right side of her lips. The treatment was performed twice a day by cutaneous applications, each of 15 minutes (total fluence 48 J/cm^2^) in an area corresponding to the parotid gland by a device emitting at 808 nm at 250 mW output power.

**Results:**

Two weeks after PBM treatment, performed at home twice a day by the patient herself without any kind of pharmacological therapy, the complete disappearing of the disease was noticed with no side effects.

**Conclusion:**

With the limitations due to a single case report and with the need of further clinical trials to confirm it, “at-home PBM” seems to represent a good and safe approach to the treatment of BP.

## 1. Introduction

Bell's palsy (BP) was first described by Sir Charles Bell (1774–1842) as an idiopathic disorder of the peripheral facial nerve causing sudden paralysis of monolateral facial muscles [1].

The incidence of BP is around 20–30 cases per 100,000 persons yearly, regardless of age and gender [2], and even if a relight of herpes simplex infection has been largely hypothesized as the main cause, this has never been demonstrated [3].

Although several patients with BP totally recover in some months, 30% of them exhibit an incomplete healing, sometimes related to social, aesthetic, or psychological problems [4].

Therefore, an immediate diagnosis and a prompt therapeutic intervention seem to be vital for achieving an optimal result [5].

Two main pharmacological kinds of drugs, steroids, and antivirals have been proposed for improving the recovery from BP, chosen on the basis of the hypothesis of its etiopathogenesis, consisting on inflammation and viral infection [6], and also for the reason that, in the saliva and facial nerve endoneural fluid of BP patients, the presence of herpes simplex virus was observed [7].

Recently, also photobiomodulation (PBM) has been suggested for the treatment of BP [8] demonstrating an immediate pain decrease as well as an antiinflammatory effect [9].

In PBM treatment, a photochemical laser-tissue interaction is involved; the energy emitted by the device is given to the mitochondria and absorbed by chromophores (endogenous porphyrins and respiratory chain components such as cytochrome c oxidase); finally, laser energy is converted into metabolic energy by the respiratory chain with the production of adenosine triphosphate (ATP); thanks to the use of parameters characterized by low-energy density, PBM can be considered free of possible side effects [10].

Its mechanism of action in BP derives both by a direct effect on nerves and by an anti-inflammatory action.

The first consists on functional activity of the injured peripheral nerve increasing, degeneration in corresponding motor neurons of the spinal cord decreasing, and axonal growth and myelinization improving [11].

The second may be provoked by levels of proinflammatory cytokines, such as interleukin-1 alpha (IL-1*α*) and IL-1 beta (IL-1*β*), reduction, and levels of anti-inflammatory growth factors and cytokines, such as basic fibroblast growth factor (bFGF), platelet-derived growth factor (PDGF), and transforming growth factor-beta (TGF-*β*), increasing [12].

Several clinical trials and case reports have demonstrated that PBM administered with adequate protocols by trained professionals may represent a good option for accelerating the recovery process in both adults and children [13–17].

Javaherian and co-authors, in a systematic review, concluded that the PBM with 830 nm and 100 mW power for a period of 6 weeks might be beneficial on recovery for the patients with subacute Bell's palsy, reporting no adverse effects during treatment and/or follow-up sessions [18].

Aghamohamdi and co-authors, in a clinical study on 30 diabetic patients with Bell's palsy, demonstrated that PBM is a safe, reliable, and proper alternative approach for the treatment of facial nerve palsy, especially in the presence of underlying conditions such as diabetes mellitus [19].

Unfortunately, the main practical problem related to PBM is the necessity for short sessions for two or three times a week and sometimes even daily [20]. To permit the patients to perform their treatment without reaching the therapist, a new laser family has recently appeared on the market; due to these devices belonging to class II of the American National Standard Institute (ANSI), they may be used directly by the patients themselves in a very simple way, thanks to the preset parameters; moreover, the use of protective goggles is not necessary, cost and size are reduced, as well as the risk of side effects and contraindications are absent; nevertheless, patients evaluation by a specialist before its use remains mandatory [21].

This case report is the first to describe the effectiveness of “at-home PBM” in the treatment of BP disease and to evidence the advantages of this technique when related to classic PBM.

## 2. Materials and Methods

### 2.1. Clinical Case

R B, a 15‐year‐old girl, came to our unit for evaluating facial paralysis on the right side. As reported by her mother, the anamnesis was totally negative not describing any important diseases, and drugs assumption and family occurrence of this condition as well as previous episodes of facial paralysis were also excluded.

**Figure 1:**
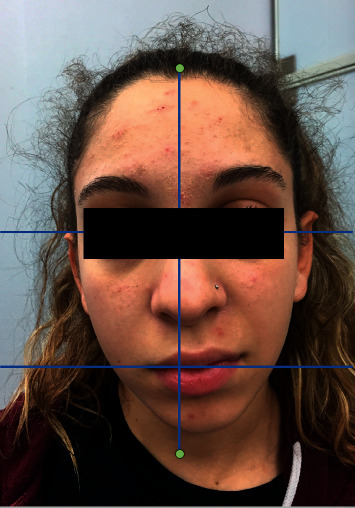
Facial observation before treatment.

The patient reported that the symptoms appeared the day before the medical examination and consisted in acute pain on the right side of the face, difficulty in biting, dripping saliva from the mouth right corner, and impossibility to obtain a complete closure of the left eye. The clinical observation revealed a facial asymmetry with edema in the right jaw, difficulty of facial movements on the right side when smiling, and asymmetry of the eyes. Due to these aspects, the diagnosis was established as an idiopathic facial paralysis, and according to the House Brackmann scale, it was classified as IV degree (moderate severe dysfunction) (Figure 1).

It was decided to treat the disease with an “self-administered PBM,” and the patient received the device as well as the instructions to correctly use it. The same day, the patient started the treatment applying the laser device (B-Cure Laser Pro, Good Energies Ltd., Haifa, Israel) on her own; this device is a class II laser according to ANSI classification, emitting in the near IR portion of the spectrum (808 nm) with a green LED aiming beam for indicating the irradiated zone of 4.5 cm^2^; its output power of 250 mW is emitted in micropulses with a frequency of 15 kHz (energy per minute of 14.4 J and fluence per minute of 3,2 J/cm^2^).

Treatment was performed twice a day by cutaneous applications, each 15 minutes (total fluence: 48 J/cm^2^) by putting the device in contact with the skin of the right side in the area corresponding to the parotid gland, as reported in other different case reports [9].

## 3. Results

**Figure 2:**
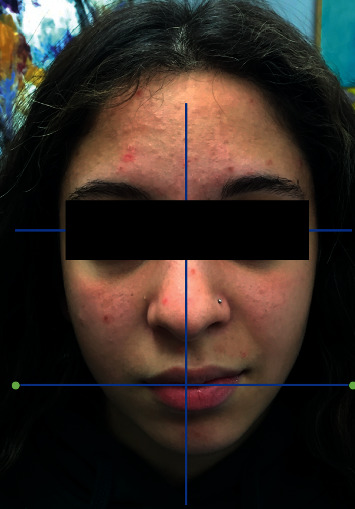
One week treatment follow-up.

One week after the first session, the facial observation revealed an important improvement of the clinical condition and the patient described a great relief of the pain; the eyes asymmetry had disappeared as well as the incapacity to close the left eye, while the asymmetry of the mouth, even if diminished, was still present (II degree of House Brackmann scale = moderate dysfunction) (Figure 2).

**Figure 3:**
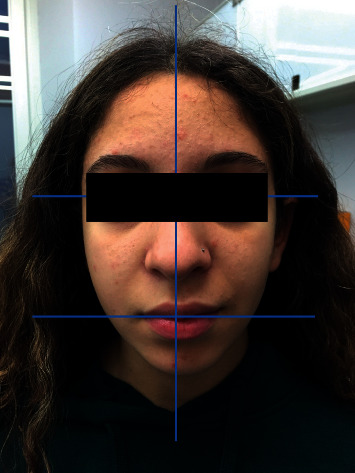
Two weeks treatment follow-up.

Two weeks later, the complete disappearing of the disease was noticed, with the normal facial functions in all areas (grade I of House Brackmann scale = normal) (Figure 3).

The patient had an important change also in her psychological sphere; she was a young girl temporarily disfigured and also worried about the prognosis of her disease which suddenly appeared compromising also her aesthetical aspect, and the rapid resolution of these signs improved her quality of life.

## 4. Discussion

Even if several cases of BP spontaneously recover some months after the initial insult, its management is focused on minimizing the risk of incomplete resolution and reducing the possibility of morbid sequelae such as facial tissues contracture, synkinesis, autonomic dysfunction, and moderate to severe facial weakness [22].

Current pharmacological approach of BP is based, as described above, on the combination of steroids and antiviral agents [23].

Recently, as PBM has shown beneficial outcome in the regeneration of peripheral nerves [24]^,^, it was proposed also in the treatment of BP to minimize the side effects of the drugs assumption [15].

PBM therapy is a noninvasive therapy defined by Anders et al. as “a form of light therapy that utilizes nonionizing forms of light;” it is a photothermal reaction which involves endogenous chromophores resulting in beneficial therapeutic phenomena, the most important being promotion of wound healing, tissue regeneration, pain and inflammation reduction, and immunomodulation [25]. The only negative aspect of PBM is represented by the need for the patient to go to the clinics two or three times weekly for performing very short sessions (five/ten minutes), which sometimes limits the compliance during the treatment.

The “at-home PBM” devices, recently introduced on the market, are very small, certified as class II lasers according to the ANSI classification and with fixed parameters; for these reasons, their utilization at home by the patients themselves is very easy and with minimal risk of overtreatment.

The energy delivered by this kind of laser devices is in the range of these suggested by the other different studies where conventional PBM was employed and, in every case, it did not exceed the fluence of 10 J, condition for avoiding the possibility to obtain an opposite effect, as established by the Arndt–Schulz rule [26].

The particularity of the “at-home PBM,” as shown in this case report, is the short time for completing the treatment and achieve the clinical result; it might be explained by the opportunity, due to the possibility to perform the treatment at home, to give the irradiation daily or twice a day, so accelerating the healing process.

## 5. Conclusion

This short case report wants to contribute to enlarge the range of “at-home PBM” clinical applications by the demonstration of the advantages of this technique: easiness of use, possibility to perform more daily sessions, no need to go to clinics for treatment, and no overtreatment risk.

With the limitations due to it is a single case report and with the necessity to confirm these results by clinical trials, “at-home PBM” seems to be a good and safe approach to the treatment of Bell's palsy.

## Data Availability

The data used to support the findings of this study are included within the article.
